# Risk Factors for Nipah Virus Infection among Pteropid Bats, Peninsular Malaysia

**DOI:** 10.3201/eid1901.120221

**Published:** 2013-01

**Authors:** Sohayati A. Rahman, Latiffah Hassan, Jonathan H. Epstein, Zaini C. Mamat, Aziz M. Yatim, Sharifah S. Hassan, Hume E. Field, Tom Hughes, Justin Westrum, M.S. Naim, Arshad S. Suri, A. Aziz Jamaluddin, Peter Daszak, the Henipavirus Ecology Research Group

**Affiliations:** Author affiliations: Veterinary Research Institute, Ipoh, Malaysia (S.A. Rahman, Z.C. Mamat, A.M. Yatim, M.S. Naim, A.A. Jamaluddin);; Universiti Putra Malaysia, Serdang, Malaysia (L. Hassan, A.S. Suri);; EcoHealth Alliance, New York, New York, USA (J.H. Epstein, T. Hughes, J. Westrum, P. Daszak);; Monash University, Selangor, (S.S. Hassan);; Queensland Primary Industries and Fisheries, Brisbane, Queensland, Australia (H.E. Field)

**Keywords:** Nipah virus, viruses, risk factors, seroprevalence, infection, distribution, bats, Pteropid bats, Pteropus vampyrus, Pteropus hypomelanus, reservoir hosts, Malaysia

## Abstract

We conducted cross-sectional and longitudinal studies to determine the distribution of and risk factors for seropositivity to Nipah virus (NiV) among *Pteropus vampyrus* and *P. hypomelanus* bats in Peninsular Malaysia. Neutralizing antibodies against NiV were detected at most locations surveyed. We observed a consistently higher NiV risk (odds ratio 3.9) and seroprevalence (32.8%) for *P. vampyrus* than *P. hypomelanus* (11.1%) bats. A 3-year longitudinal study of *P. hypomelanus* bats indicated nonseasonal temporal variation in seroprevalence, evidence for viral circulation within the study period, and an overall NiV seroprevalence of 9.8%. The seroprevalence fluctuated over the study duration between 1% and 20% and generally decreased during 2004–2006. Adult bats, particularly pregnant, with dependent pup and lactating bats, had a higher prevalence of NiV antibodies than juveniles. Antibodies in juveniles 6 months–2 years of age suggested viral circulation within the study period.

## Abstract 2

Infection rates may be higher during pregnancy and lactation.

Nipah virus (NiV) disease emerged in Peninsular Malaysia during September 1998–April 1999 and resulted in 105 human deaths (≈40%) and compulsory culling of 1.1 million pigs (*1*). NiV disease has been successfully controlled in Malaysia. However, elsewhere in Bangladesh and India, 12 NiV outbreaks have since occurred (*2*). Pteropid bats (family Pteropodidae, genus *Pteropus*) were the most likely reservoir host of the virus, and evidence of NiV in these bats had been consistently found in populations across southern Asia (*3**–**6*) and Africa (*7*) despite an absence of outbreaks in some areas.

Characteristics of bats that promote their competency as a natural host and reservoir for many emerging pathogens from evolutionary, ecologic, sociobehavioral, and immunologic perspectives are progressively being reported (*8**–**11*). In Malaysia, previous surveillance work suggest 2 pteropid species, *Pteropus hypomelanus* (variable flying fox) and *P*. *vampyrus* (large flying fox) bats, as reservoir hosts for NiV (*12*). *P. hypomelanus* bats reside on offshore islands along the eastern (n = 14) and western (n = 4) coasts of the peninsula (*13*). NiV was first isolated from pooled urine samples from these bats on the island of Pulau Tioman off the eastern coast of the state of Pahang (*14*). *P. vampyrus* bats, the pteropid species identified during the first NiV disease outbreak location in 1998 (*3*), are anthropogenic-susceptible bats residing in remote and inaccessible areas such as mangroves and dense forests (*15*). *P. vampyrus* bats roost mainly on the mainland but may have focal transitory points on surrounding islands as they travel (*16**,**17*).

Our recent follow-up work on a cohort of *P. vampyrus* bats (*18**,**19*) showed a possible NiV recrudescent event leading to horizontal viral transmission to other bats in the colony. The findings further elucidated maintenance and transmission dynamics of the virus within and among roosts, colonies, and the bat metapopulation. In this report, we present results of 2 studies conducted concurrently to determine the geographic extent and prevalence of NiV-neutralizing antibody for *P. vampyrus* and *P. hypomelanus* bats, and to identify the sexual and reproductive maturity determinants for NiV seropositivity in the wild.

## Materials and Methods

### Study Design and Study Populations

#### Cross-sectional Study of NiV (Distribution Study)

**Figure 1:**
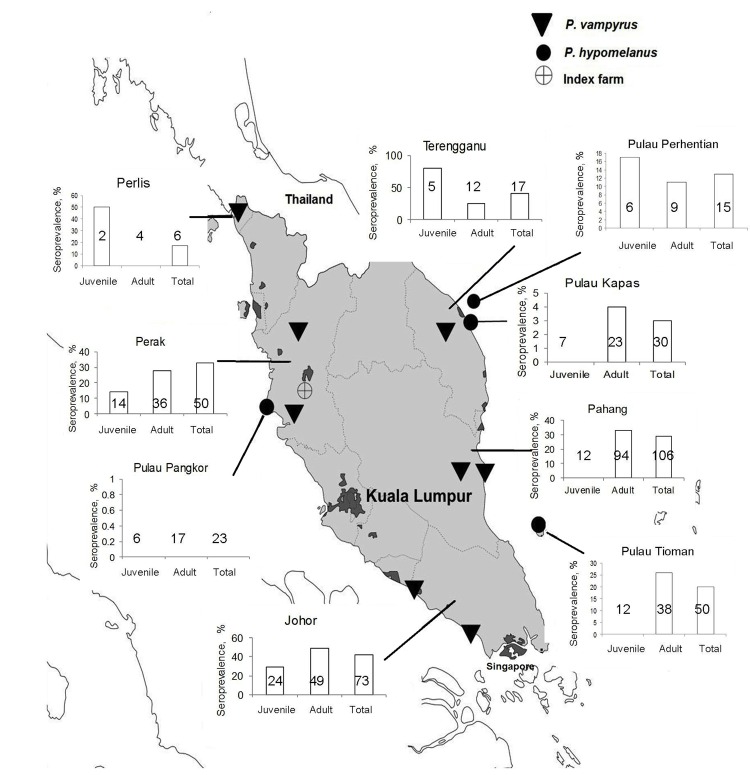
Trapping sites for *Pteropus hypomelanus* and *P. vampyrus* bats and seroprevalence of Nipah virus in 8 sites, Peninsular Malaysia, January 2004–September 2006. Values in the small graphs indicate number of positive samples.

During March 2004–May 2007, we sampled *P. hypomelanus* bat colonies from several roost sites in Pulau Kapas and Pulau Perhentian on the northeast coast of Terengganu, Pulau Tioman on the southeast coast of Pahang, and Pulau Pangkor on the northwest coast of Perak. We also sampled *P. vampyrus* bats in the states of Perlis; Perak (Teluk, Memali, and Lenggong); Terengganu (Kampung Alor Lek, Setiu, and Kuala Berang); Pahang (Tanjung Agas, Pasir Panjang, and Ganchong); and Johor (Benut and Kesang) (Figure 1). We determined these sites using published information on pteropid roost locations (*15*), reports from the Malaysia Department of Parks and Natural Resources authority (PERHILITAN), information from local hunters and residents, and field observations. A complete list of observed roost sites was published by Epstein et. al. (*16*). Colonies were selected for this study on the basis of observed presence of bats, accessibility for bat capture, and roost size (e.g., number of bats). A sample size of 35 bats was targeted at each location or for each sampling effort to be able to detect a NiV-seropositive bat given a minimum prevalence of 10% with 10% precision at a confidence level of 95% (*20*).

Permission for the study was granted by PERHILITAN, and Institutional Animal Care and Use Committee approval was obtained from the Wildlife Trust Institutional Animal Care and Use Committee (New York, NY, USA). A special permit to trap and humanely kill the bats was obtained from PERHILITAN. Bats were captured nonrandomly by using 2 methods: opportunistic sampling of hunted bats (hunters were not solicited or incentivized to hunt bats for this study) and mist nets.

Hunted bats were sampled at the site of hunting, and bats were attributed to the location of the nearest known roost. Blood samples were collected by cardiac puncture, and the kidneys were harvested. The blood, urine, and kidney samples were processed as described (*18*). Bats captured in mist nets were extracted from the nets immediately after capture and anesthetized before sampling by using medetomidine, a combination of medetomidine/ketamine (*21*), or isoflurane gas. Blood (3 mL) was collected from the cephalic vein or brachial vein and placed into serum-separator tubes (Vacutainer; Becton Dickinson, Franklin Lakes, NJ, USA). Sterile cotton swabs were used to collect oropharyngeal and urogenital samples. All samples were stored in liquid nitrogen at −190°C) and transported to the Veterinary Research Institute in Ipoh, Perak, Malaysia.

For each bat hunted or captured, information on the date of sampling, species, location, sex, reproductive status, and estimated age was recorded. We assigned each bat an age category of adult (secondary sexual characteristics visible/reproductive), juvenile (no observable secondary sexual characteristics, not pregnant, dental wear characteristics), or pup (dependent and attached to its dam) (*3*). We categorized the reproductive status as pregnant, carrying a pup (adult females with attached pup), nursing (adult females without attached pup but with evidence of lactation), or dry (not in any of the previous categories) using published criteria (*18*). The total number of bats sampled for each pteropid species is shown in Tables 1 and 2. Bats captured in mist nets were released at the site of capture after recovery from anesthesia.

**Table 1:** *Pteropus vampyrus* bats with Nipah virus–neutralizing antibody titers, Peninsular Malaysia, January 2004–October 2006*

Location	Sex	Age	No.	SNT titer	No. positive	% Seroprevalence (95% CI)
Perlis, n = 6	M	Adult	4	<8	0	16.7 (1.1–58.2)
		Juvenile	0	NA	NA	
		Pup	0	NA	NA	
	F	Adult	0	NA	NA	
		Juvenile	2	<8	1	
		Pup	0	NA	NA	
Perak, n = 50	M	Adult	18	<8–32	3	24 (14.1–37.5)
		Juvenile	11	<8–64	2	
		Pup	0	NA	NA	
	F	Adult	18	<8–512	7	
		Juvenile	3	<8	0	
		Pup	0	NA	NA	
Terengganu, n = 18	M	Adult	6	<8–128	1	38.9 (20.2–61.5)
		Juvenile	5	<8–64	4	
		Pup	0	NA	NA	
	F	Adult	6	<8–16	2	
		Juvenile	0	NA	NA	
		Pup	1	<8	0	
Johor, n = 73	M	Adult	23	<8–128	12	42.4 (31.7–53.9)
		Juvenile	16	<8–128	6	
		Pup	0	NA	NA	
	F	Adult	26	<8–128	12	
		Juvenile	8	<8–16	1	
		Pup	0	NA	NA	
Pahang, n = 106	M	Adult	44	<8–1,024	10	29.2 (21.4–38.5)
		Juvenile	9	<8	0	
		Pup	0	NA	NA	
	F	Adult	50	<8–1,024	21	
		Juvenile	3	<8	0	
		Pup	0	NA	NA	
Overall, N = 253		Adult	193	<8–1,024	0	32.8 (27.3–38.8)
		Juvenile	66	NA	14	
		Pup	1	NA	68	

*SNT, serum neutralization test; NA, not applicable (no sample).

**Table 2:** *Pteropus hypomelanus* bats with Nipah virus–neutralizing antibody titers, Peninsular Malaysia, January 2004–October 2006*

Location	Sex	Age	No.	SNT titer	No. positive	% Seroprevalence (95% CI)
Pulau Pangkor, n = 23	M	Adult	13	<8	0	0 (0–16)
		Juvenile	4	<8	0	
		Pup	0	NA	NA	
	F	Adult	4	<8	0	
		Juvenile	2	<8	0	
		Pup	0	NA	NA	
Pulau Perhentian, n = 15	M	Adult	2	<8–128	0	13.3 (2.5–39.1)
		Juvenile	1	<8	1	
		Pup	0	NA	NA	
	F	Adult	7	<8–64	1	
		Juvenile	5	<8	0	
		Pup	0	NA	NA	
Pulau Kapas, n = 29	M	Adult	7	<8	0	3.3 (0.01–18.1)
		Juvenile	0	NA	NA	
		Pup	0	NA	NA	
	F	Adult	15	<8	1	
		Juvenile	7	<8–32	0	
		Pup	0	NA	NA	
Pulau Tioman, n = 50	M	Adult	27	<8–128	6	20 (11.1–33.2)
		Juvenile	4	<8	0	
		Pup	0	NA	NA	
	F	Adult	11	<8–256	4	
		Juvenile	8	<8	0	
		Pup	0	NA	NA	
Overall, N = 117		Adult	73	<8–256	13	11.1 (6.5–18.2)
		Juvenile	31	NA	0	
		Pup	0	NA	0	

*SNT, serum neutralization test; NA, not applicable (no sample).

We performed descriptive analysis to describe the seroprevalence on the basis of species of bats and locations. Differences among positive antibody titers (range <8–1,025) between species were tested by using the Mann-Whitney U test. Comparison of the seroprevalence rates between the 2 pteropid species was performed by using the χ^2^ test. When there were sufficient data to perform the analysis, several factors were examined for its association to NiV seropositivity by using the χ^2^ test.

#### Longitudinal Study on Risk Factors for NiV (Risk Factor Study)

We performed a longitudinal study on a population of *P. hypomelanus* bats in Pulau Tioman in which 50 bats from the same colony were captured by using mist nets and sampled approximately every 6–8 weeks during January 2004–October 2006. Monsoonal rains prevented access to the island of Pulau Tioman during December–February, which extended the interval between some sampling points. Captured bats were tagged by using a thumb band or implantable microchip (Avid Identification Systems, Inc., Norco, CA, USA) and unique identification numbers to ensure that sampling was not repeated on the same bat. The bats were anesthetized by using ketamine and xylazine (*22*). Data, blood, and swab samples were collected as described in the previous section.

We investigated associations between serostatus (positive or negative) and each of the hypothesized risk factors (sex, age, reproductive categories, time) using the χ^2^ test or Fisher exact test when the χ^2^ test was not appropriate. Our previous study (*18*) suggested a correlation between the serologic status of pups and their dams. Therefore, serologic data for *P. hypomelanus* pups were excluded from further analysis. Logistic regression with generalized estimating equations was used to analyze longitudinal data to control for the effect of clustering, assuming that bats sampled between various times were from the same population. To compare the categories across sexual and reproductive maturity, we reclassified the data on the basis of sex (male and female) and reproductive maturity (juvenile, adult, dry, pregnant, carrying a pup, and nursing) and renamed the variable the sexual and reproductive maturity factor. The logistic regression analysis includes the sexual and reproductive maturity categories and sampling time for the bats. All hypothesis testing was 2-sided, with α = 0.05, and was performed by using SPSS version 19 (SPSS Inc., Chicago, IL, USA).

### Laboratory Analysis

Plasma-neutralizing antibodies against NiV were measured by using the serum neutralization test (SNT) (*23*) with plasma diluted 1:2–1:1,024. A titer ≥8 was considered a positive titer for specific antibody against NiV because serum samples were usually toxic to Vero cells at higher concentrations (i.e., 1:2 or 1:4). Virus isolation was attempted by using rabbit kidney and Vero cells in a biosafety level 3 facility until the third passage before a sample was considered negative (*23*). According to the Malaysian Government Act on Control and Prevention of Infectious Diseases 1988 (revised May 25, 2006), NiV is categorized in risk group 3, which enables the virus to be handled in a biosafety level 3 facility. Any tissue culture with an NiV-like cytopathic effect was confirmed by using a PCR as described (*24*). All laboratory diagnostics (SNT, virus isolation, and PCR) were conducted at the Veterinary Research Institute in Ipoh.

## Results

### Distribution of NiV

#### *P. vampyrus* Bats

NiV-neutralizing antibodies were detected in bats at all locations (Table 1). Overall, 82 (32.8%) of 253 bats were seropositive. We found no significant difference in seroprevalence rates in bats from the 5 states (p = 0.213, by Fisher exact test). NiV-neutralizing antibody titers ranged from <8 to 1,024 (median <8), and among seropositive bats, the median titer was 64. All culture and PCR results were negative. Among pups, juvenile, and adult bats, 0% (0/1), 25% (14/56), and 35.2% (68/193), respectively, were seropositive (Table 1). Age, sex, and female reproductive status were examined for their effects on serostatus of *P. vampyrus* bats. Univariate analysis showed that age and sex were not associated with seroprevalence of NiV. However, among nursing bats, a higher risk for NiV seropositivity was observed than in other adult females (Table 3).

**Table 3:** Univariate analysis of independent variables and Nipah virus serostatus of 2 *Pteropus* bat species surveyed in a cross-sectional survey (without pup data), Peninsular Malaysia, January 2004–October 2006*

Species and risk factor	No. positive/no. tested (%)	p value†	OR (95% CI)
*P. vampyrus*			
Age			
Juvenile	14/56 (25)	Referent	1.00
Adult	68/193 (35.2)	0.151	1.63 (0.83–3.19)
Sex			
M	38/135 (28.1)	Referent	1.00
F	44/114 (38.6)	0.081	1.6 (0.94–2.73)
Female reproductive status			
Dry	21/58 (36.2)	Referent	1.00
Pregnant	6/14 (42.3)	0.645	1.32 (0.40–4.33)
Carrying a pup	3/7 (42.9)	0.731	1.32 (0.27–6.48)
Nursing	11/17 (64.7)	0.042	3.23 (1.04–9.99)
Juvenile	3/18 (16.7)	0.130	0.35 (0.09–1.36)
*P. hypomelanus*			
Age			
Juvenile	0/31 (0)	NA	NA
Adult	13/86 (15.1)	NA	NA
Sex			
M	7/58(12.1)	Referent	1.00
F	6/59 (10.2)	0.744	0.85 (0.26–2.62)
Female reproductive status			
Dry	2/23 (8.7)	Referent	1.00
Pregnant	4/14 (28.6)	0.112	4.20 (0.65–26.89)
Carrying a pup	NA	NA	NA
Nursing	NA	NA	NA
Juvenile	0/22 (0)	NA	NA

*OR, odds ratio; NA, not applicable. †By χ^2^ test, Fisher exact test, or simple logistic regression.

#### *P. hypomelanus* Bats

Of 119 plasma samples collected from island sites, 2 were not used because of inadequate amounts of plasma. Of the remaining 117, 13 (11.1%) were seropositive. NiV antibodies were detected in 13 (13.8%) of 94 bats from islands off the east coast (Pulau Perhentian, Pulau Kapas, Pulau Tioman) of Peninsular Malaysia. Titers ranged from <8 to 256 (median <8); among seropositive bats, the median titer was 32. Samples from Pulau Pangkor (n = 24) were negative for NiV-neutralizing antibodies. All culture and PCR results were negative. Pups were not captured, but among juvenile and adult bats, 0% (0/31) and 11.1% (13/73), respectively, were seropositive (Table 2). Univariate analysis showed that sex was not associated with NiV seropositivity. Stratified data for various reproductive categories were sparse and lacked power for meaningful analysis (Table 3).

#### Comparison of *P. vampyrus* and *P. hypomelanus* Bats

The antibody titer difference between *P.* *vampyrus* and *P. hypomelanus* bats was significant (p<0.001, by Mann-Whitney U test. The difference in seroprevalence for NiV between *P. hypomelanus* (13/117, 11.1%) and *P. vampyrus* (82/253, 32.8%) bats was significant (χ^2^ 19.54, p<0.001), and risk for a seropositive reaction to NiV was 3.9× higher for *P. vampyrus* bats than for *P. hypomelanus* bats.

### Longitudinal Study of NiV *P. hypomelanus* Bats

**Figure 2:**
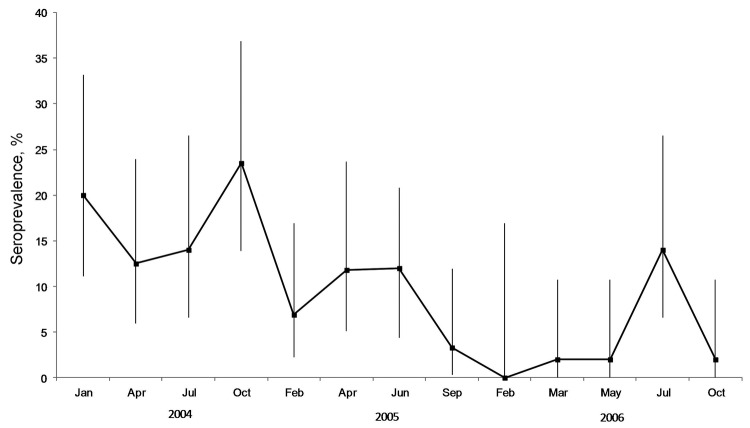
Seroprevalence of Nipah virus among *Pteropus hypomelanus* bats in Pulau Tioman, Peninsular Malaysia, January 2004–September 2006. Error bars indicate 95% CIs.

Characteristics of bats sampled are shown in Table 4. All P. *hypomelanus* bat samples had negative culture and PCR results. The overall NiV seroprevalence for *P. hypomelanus* bats from Pulau Tioman was 9.8%. Differences in seroprevalence between sampling times were significant (p<0.001). The highest seroprevalence rate was in 2004, which then waned in 2005 and 2006 (Figure 2).

**Table 4:** Characteristics of *Pteropus hypomelanus* bats tested for Nipah virus, Pulau Tiomanm, Peninsular Malaysia, January 2004–October 2006*

Sex	Age group	No.	Reproductive status	No.	SNT titer range	No. (%) seropositive	% Seroprevalence (95% CI)
Male, n = 407	Adult	314	NA	NA	<8–512	39 (12.4)	10.80 (8.18–14.27)
	Juvenile	81	NA	NA	<8–32	5 (6.1)	
	Pup	12	NA	NA	<8	0	
Female, n = 243	Adult	124	Dry	62	<8–256	4 (6.5)	8.20 (5.30–12.4)
			Pregnant	19	<8–256	4 (21.1)	
			Attached	20	<8–256	5 (25)	
			Nursing	23	<8–128	4 (17.4)	
	Juvenile	113	NA	NA	<8–64	2 (1.8)	
	Pup	6	NA	NA	<8–64	1 (16.6)	
Overall, N = 650	NA	650	NA	NA	<8–512	64	9.80 (7.77–12.39)

*SNT, serum neutralization test; NA, not applicable.

The seroprevalence of NiV in adult bats (12.8%) was significantly higher than in juveniles (3.7%; p<0.001), and adults were ≈4× more likely to be seropositive (odds ratio 3.9). Seroprevalence was not significantly different between male (11.1%) and female bats (8%; p = 0.258). However, when data for female bats were examined by their reproductive categories, a significant difference (p<0.001) was observed. Bats that were pregnant, carrying a pup, and lactating were 3.9×, 4.8×, and 3.0× more likely to be seropositive than adult female bats that did not show these features (Table 5).

**Table 5:** Univariate analysis of independent variables and Nipah virus seropositivity (without pup data) in 632 samples from *Pteropus hypomelanus* bats surveyed in Pulau Tioman, Peninsular Malaysia, January 2004–October 2006

Risk factor	No. positive/no. tested (%)	p value*	Odds ratio (95% CI)
Sampling time			
Jan 2004	10/50 (20)	Referent	1.00
Apr 2004	7/50 (14)	0.297	0.57 (0.20–1.64)
Jul 2004	7/50 (14)	0.427	0.65 (0.23–1.88)
Sep 2004	12/51 (6.3)	0.668	1.23 (0.48–3.18)
Feb 2005	4/54 (6.9)	0.052	0.29 (0.08–1.01)
Apr 2005	5/41 (6.9)	0.262	0.53 (0.18–1.59)
Jun 2005	6/50 (12)	0.280	0.54 (0.18–1.63)
Sep 2005	2/61 (3.3)	0.013	0.13 (0.03–0.65)
Feb and Mar 2006†	1/71 (1.4)	0.007	0.05 (0.01–0.45)
May 2006	1/50 (2)	0.019	0.08 (0.01–0.66)
Jul 2006	7/50 (14)	0.427	0.65 (0.22–1.88)
Oct 2006	1/50 (2)	0.019	0.08 (0.10–0.66)
Age			
Juvenile	7/194 (3.7)	<0.001	1.00
Adult	56/438 (12.8)		3.91 (1.75–8.75)
Sex			
M	44/395 (11.1)	0.213	1.00
F	19/237 (8.1)		0.69 (0.39–1.22)
Female reproductive status			
Dry	4/65 (6.5)	Referent	1.00
Pregnant	4/19 (21)	0.077	3.87 (0.86–17.29)
Carrying a pup	5/20 (25)	0.031	4.83 (1.15–20.24)
Nursing	4/23 (17.4)	0.139	3.05 (0.69–13.40)
Juvenile	2/113 (1.8)	0.128	0.26 (0.05–1.47)

*By χ^2^ test, Fisher exact, or simple logistic regression. †Data were combined to enable analysis.

Although our data were not sufficiently powered to detect significant differences among various sex and reproductive maturity categories (sample size was not large enough within each category level), when we controlled for the effect of time, the likelihood of seropositivity to NiV among pregnant, carrying, and nursing females remained higher than other female bats, and the highest risk was observed among those females carrying pups. Juvenile female bats were the least likely to have detectable NiV antibodies compared with other bats. Most seropositive cases appeared to cluster in 2004 and decreased toward the end of the study (Table 6).

**Table 6:** Risk factors for Nipah virus seropositivity (without pup data) in *Pteropus hypomelanus* bats sampled in Pulau Tioman, Peninsular Malaysia, January 2004­–October 2006*

Variable	β	SE	p value	OR (95% CI)
Time				
Jan 2004	Referent	Referent	Referent	1.00
Apr 2004	−0.58	0.57	0.310	0.55 (0.17–1.72)
Jul 2004	0.52	0.59	0.373	1.69 (0.53–5.43)
Sep 2004	0.38	0.51	0.457	1.46 (0.53–3.97)
Feb 2005	−1.31	0.62	0.034	0.26 (0.07–0.90)
Apr 2005	−1.17	0.78	0.135	0.30 (0.06–1.44)
Jun 2005	−0.44	0.59	0.459	0.64 (0.19–2.07)
Sep 2005	−1.54	0.76	0.045	0.21 (0.04–0.96)
Feb and Mar 2006†	−2.59	1.09	0.018	0.07 (0.00–0.63)
May 2006	−2.13	1.09	0.051	0.11 (0.01–1.00)
Jul 2006	−0.32	0.56	0.564	0.72 (0.24–2.17)
Oct 2006	−2.47	1.08	0.022	0.08 (0.01–0.70)
Sexual and reproductive maturity				
Adult males	Referent	Referent	Referent	1.00
Juvenile males	−0.78	0.52	0.132	0.45 (0.16–1.27)
Dry females	−0.84	0.53	0.114	0.42 (0.14–1.22)
Pregnant females	0.50	0.62	0.421	1.65 (0.48–5.61)
Carrying a female pup	1.24	0.69	0.074	3.48 (0.88−13.6)
Nursing females	0.50	0.63	0.427	1.65 (0.47–5.74)
Juvenile females	−2.34	0.70	0.001	0.09 (0.02–0.38)

*β, estimated coefficient; OR, odds ratio. †Data were combined to enable analysis.

Anthropic disturbance to roosting sites during our study resulted in dispersal of *P. hypomelanus* bats to smaller (<20), more disparate cohorts that roosted higher in the trees and finally relocation to another site on Pulau Tioman, ≈6 km from the previous site. We believe roost size affected seroprevalence rates and thus reduced risks for seropositivity, as observed toward the end of the study.

## Discussion

All bats captured during the study, including NiV-seropositive bats, appeared healthy, which was consistent with observations from experimental infections of pteropid bats with NiV (*25**,**26*). Our cross-sectional survey found that seroprevalences of NiV in *P. vampyrus* and *P. hypomelanus* bats were 32.8% and 11.1%, respectively, which differed from results of a study Yob et al. (*12*) after the first NiV outbreak (17% vs. 31%, respectively). Although both studies support the hypothesis that *Pteropus* spp. are the natural reservoir for NiV in Malaysia, we believe that this difference may be attributed to our study having a larger sample size for each species and a wider geographic sampling scale, as well as potential differences among the diagnostic assays used.

The presence of NiV-seropositive *P. vampyrus* bats across Peninsular Malaysia was expected because satellite telemetry studies by Epstein et al. (*16**)* and Breed et al. (*17*) showed that *P. vampyrus* bats are highly mobile, moving beyond state and national borders and making contact with other conspecifics across the region probable. These findings are consistent with the findings of studies of Hendra virus in Australia and NiV in Bangladesh, in which evidence for circulation has been observed across a broad expanse of the home range of each pteropid species (*27**–**29*). The higher seroprevalence of NiV among *P. vampyrus* bats than among *P. hypomelanus* bats suggests that exposure to NiV is more common among the *P. vampyrus* bats, which is consistent with higher rates of NiV seroprevalence among juvenile *P. vampyrus* bats than among juvenile *P. hypomelanus* bats. These findings suggest that viral circulation and exposure are probably more common among *P.* *vampyrus* bats.

We believe that the greater connectivity of *P. vampyus* bats among colonies in the region create a metapopulation structure in which there is more opportunity for virus to circulate by migration, resulting in a higher rate of exposure. Island bats have limited connectivity (*30*). Thus, herd immunity tends to wane over time, creating a relatively lower seroprevalence (*11*). This phenomenon may be illustrated by our survey of *P. hypomelanus* bats on Pulau Pangkor, in which we did not detect any seropositive bats. We sampled 24 bats, which would have enabled us to detect a seropositive bat, given a prevalence >10% with 95% confidence. The fact that we did not detect any seropositive bats suggests a lower seroprevalence, which could also be attributed to the relatively high degree of urbanization on Pulau Pangkor and consequently smaller colonies and a decreased seroprevalence.

The longitudinal study of *P. hypomelanus* bats showed that prevalence of neutralizing antibodies to NiV fluctuated during the study (range 1%–20%) and, although unpredictable, seroprevalence generally waned in the final 2 years (Figure 2). We did not observe any consistent and discernible seasonal seroprevalence pattern. However, other studies have suggested periodic patterns of antibody prevalence that are connected to the reproductive cycle of bats for other viruses such as Hendra virus (*31*), filovirus, and lyssavirus (*8**,**9*). With the exception of the first NiV outbreak in Malaysia, other studies have linked NiV outbreaks to bat reproductive seasons (*32**–**34*). We believe that the waning seroprevalence during our study is partly explained by anthropic disturbances that occurred at the original study site in Pulau Tioman, which resulted in relocation and dispersal of the original colony into smaller roost sizes. In addition to waning immunity in bats, smaller roost size decreases viral transmission to other susceptible bats, resulting in decreasing seroprevalence over time. The lack of viral isolation from any sample collected during our studies may have been caused by a low incidence of viral shedding or low viral excretion doses within the colony, which is supported by studies of henipaviruses (*1**,**4**,**5**,**18**,**35*).

Sex was not a risk factor for NiV exposure, which is consistent with results of other studies (*28**,**31**,**36*). However, when we stratified the analysis on the basis of sexual and reproductive maturity of the bats, we found that female bats that were pregnant, had an attached pup, and were lactating had a consistently higher likelihood of exposure to NiV than adult males or dry adult females. Analysis of data from a cross-sectional survey of bats of both species suggests an increasing risk for exposure to NiV when female bats were pregnant or lactating. This finding was strengthened in the longitudinal study because female bats that were pregnant, had an attached pup, or were nursing had a higher risk for NiV when we controlled for the potential confounding effect of sampling time (or seasonality).

Among mammals, *Pteropus* spp. bats are known to carry their pups for <3 months (*30*) after birth and continue to nurse for <4 months after the pup is independent. Pregnancy and lactation are the most metabolically demanding periods of mammalian life (*37*). Therefore, the cumulative stress from reproductive activities, followed by physical exertion from carrying an attached pup to lactation, may have led to the increased risk for NiV infection among this group of bats, consistent with the findings of Plowright et al. (*31*). We speculate that NiV spillover events are most likely to occur during these periods.

Our study has several limitations, including sampling bias, which results from the nonrandom sampling technique used. Sample numbers were often suboptimal because of extreme difficulty in catching pteropid bats. In addition, there are major challenges associated with interpretation of serologic data in wildlife populations. Although we detected differences in the prevalence of neutralizing antibodies between species and increased risk among bats in different reproductive categories, there is still little known about the timing of actual infection or the duration of NiV antibodies in bats. One technique used to overcome this difficulty was to examine age-stratified serologic data, which has been used in similar epidemiologic studies (*31*). *P. hypomelanus* bats reach sexual maturity at ≈12 months of age (*30*) and *P. vampyrus* at 24 months of age (*38*), which enabled us to infer that NiV antibodies in weaned juveniles (≈6–24 months of age) may indicate recent viral circulation. Our previous study among captured *P. vampyrus* bats demonstrated horizontal transmission of NiV from an adult to juvenile bats within the same colony after a brief shedding episode (*18*). Our finding of juvenile *P. hypomelanus* bats with antibodies to NiV during the longitudinal study support the possibility that virus had been circulating in this population within the lifetime of the juvenile, assuming that the juvenile bats were old enough to have lost maternal antibodies.

Our finding of neutralizing antibodies to NiV in both species of *Pteropus* bats at almost all locations we studied in Malaysia, coupled with isolation of NiV from these bats by our group and another group, strengthened the theory that NiV is enzootic in both *Pteropus* bat species, and that these species serve as the natural reservoir for NiV in Malaysia. Longitudinal surveys of *P. hypomelanus* bats suggest size and colony density may cause lower seroprevalence, and female bats that were pregnant, carrying a pup, and lactating generally had higher rates of NiV exposure than males or nonpregnant adult females, which lend further support to the hypothesis that infection rates may be higher during periods of pregnancy and lactation. Further study of NiV infection and shedding rates in pteropid bats will help elucidate seasonal and intracolonial viral dynamics.
